# Urinary Tract Infection and Associated Factors among Pregnant Women Receiving Antenatal Care at a Primary Health Care Facility in the Northern Region of Ghana

**DOI:** 10.1155/2023/3727265

**Published:** 2023-06-02

**Authors:** Ezekiel K. Vicar, Samuel E. K. Acquah, Williams Wallana, Eugene D. Kuugbee, Emmanuel K. Osbutey, Abigail Aidoo, Emmanuel Acheampong, Gloria Ivy Mensah

**Affiliations:** ^1^Department of Clinical Microbiology, School of Medicine, University for Development Studies, Tamale, Northern Region, Ghana; ^2^Department of Infectious Diseases, School of Allied Health Science, University for Development Studies, Tamale, Northern Region, Ghana; ^3^School of Medical Sciences, C. K. Tedam University of Science and Technology, Navrongo, Upper East. Region, Ghana; ^4^Department of Anatomy, School of Medicine, University for Development Studies, Tamale, Northern Region, Ghana; ^5^Department of Midwifery and Women's Health, School of Nursing and Midwifer, University for Development Studies, Tamale, Northern Region, Ghana; ^6^Department of Molecular Medicine, School of Medicine and Dentistry, Kwame Nkrumah University of Science and Technology, Kumasi, Ghana; ^7^School of Health and Medical Sciences, Edith Cowan University, Joondalup, Australia; ^8^Department of Bacteriology, Noguchi Memorial Institute for Medical Research, University of Ghana, Legon Greater Accra Region, Accra, Ghana

## Abstract

Urinary tract infection (UTI) is frequently encountered during pregnancy and is associated with adverse maternal, fetal, and neonatal effects. However, very little information is available on the prevalence of UTI among pregnant women in the northern part of Ghana, a region with a high birth rate. This study employed a cross-sectional analysis of the prevalence, antimicrobial profile, and risk factors associated with UTI in 560 pregnant women attending primary care for antenatal check-ups. Sociodemographic obstetrical history and personal hygiene information were obtained using a well-structured questionnaire. Afterward, clean catch mid-stream urine samples were collected from all participants and subjected to routine microscopy examination and culture. Of 560 pregnant women, 223 cases (39.8%) were positive for UTI. There was a statistically significant association between sociodemographic, obstetric, and personal hygiene variables and UTI (*p* < 0.0001). *Escherichia coli* (27.8%) was the commonest bacterial isolate followed by CoNS (13.5%) and *Proteus species* (12.6%). These isolates exhibited greater resistance to ampicillin (70.1–97.3%) and cotrimoxazole (48.1–89.7%) but were fairly susceptible to gentamycin and ciprofloxacin. Gram-negative resistance to meropenem was up to 25.0%, and Gram positives resistance to cefoxitin and vancomycin was up to 33.3% and 71.4% respectively. The current findings extend our knowledge of the high frequency of UTIs and associated risk factors in pregnant women with *E. Coli* being the predominant and usual isolate. Variation existed in the resistance pattern of isolates to various drugs, underscoring the need to perform urine culture and susceptibility before treatment.

## 1. Introduction

Pregnancy causes several physical, hormonal, and functional changes in the urinary tract [1]. This increases urine stasis and the ascending of microbially contaminated urine from the bladder into the ureters, causing urinary tract infection (UTI) [1, 2]. The changes in the urinary tract and immunological changes associated with pregnancy, along with an already short urethra predispose women to UTI [3, 4]. Maternal UTI is the commonest nonintestinal infection in pregnant women globally, and it is a major health problem affecting up to 20% of expectant mothers [5, 6]. UTIs can either be symptomatic or asymptomatic and are linked with serious obstetric complications and poor maternal and neonatal outcomes such as preterm birth, low birth weight, stillbirth, sepsis, maternal anaemia, pyelonephritis, preeclampsia, amnionitis, and neonatal deaths [7–9]. Therefore, early diagnosis and treatment are timely and critical to prevent complications [10].

The prevalence of UTI among pregnant women varies among study populations. In Africa, it ranges from 11.6% to as high as 75% [11, 12]. In Ghana, the prevalence of UTI among pregnant women is represented by fragmented and small-size pocketed studies with a reported prevalence range of 42.8 to 56.5% [6, 13–16]. Approximately the most common reported pathogenic cause of UTI are members of the *Enterobacteriaceae* family which are responsible for 80% to 95% of all cases, of which *Escherichia coli* is the most frequently isolated bacteria [17, 18].

The severity of an UTI is usually influenced by the virulence of the bacteria and the susceptibility of the host. Antibiotics such as ceftazidime, amoxicillin, cefoxitin, penicillin, and norfloxacin have been widely used to empirically treat clinically examined individuals with UTI. However, over time, inappropriate prescriptions for the treatment of UTIs along with their widespread use have caused bacteria to mutate and develop drug resistance. A UTI can be caused by bacteria resistant to common antibiotics. This makes treating UTIs more difficult and raises the risk of complications, underscoring the need for using antibiotics correctly and identifying the best drug [6, 11, 19–23].

The most reliable tool for diagnosing UTI is urine culture, as it helps to detect and quantify the pathogen causing the infection [24]. In Ghana, as it is with most developing countries, routine culture tests are not done for persons receiving antenatal care. Instead, a urine dipstick analysis is done which is woefully inadequate to assess UTI in pregnant women [19]. Moreover, several factors, including parity, gravidity, gestational age, history of UTI, diabetes, anaemia, socio-economic status, educational status, sexual activity, and catheterization have been associated with an increased risk of UTI during pregnancy [25–27].

In cultured urine samples taken from pregnant women visiting the Kumbungu health center, we observed an unusually high occurrence of proteinuria, haematuria, and positive bacterial growth. These observations, along with the limited information on the prevalence of UTIs in the northern part of Ghana, raise serious concerns regarding the likelihood that pregnant in the Kumbungu district seeking antenatal care would contract UTIs. Thus, the aim of this study was to determine the prevalence of UTI in pregnant women receiving antenatal care in a primary health facility in Kumbungu, Northern Ghana, as well as the associated risk factors and the antibiotic susceptibility profile of the implicated aetiological agents.

## 2. Material and Methods

### 2.1. Study Design and Setting

We conducted a cross-sectional study at the Reproductive and Child Health (RCH) unit of the Kumbungu health centre between 21st January 2019 to 30th November 2019. This facility serves predominantly the rural dweller of the Kumbungu District. It is located in the Northern region of Ghana. The district has a population of 110,586 residents of which 55,295 being women and a population density of 73.75/km^2^ [28].

### 2.2. Study Population and Selection of Participants

The targeted study population was pregnant women attending an antenatal care clinic. By employing a random sampling technique, a total of 560 pregnant women who visited the antenatal clinic were recruited. Depending on the total number of clinic attendees per day, between 30 and 40 pregnant women were randomly recruited weekly until the sample size was achieved. We numbered 30 small pieces of cardboard and placed them in a box and pregnant women who met our selection criteria were asked to pick one. This was done till the number of pregnant women for the day was reached. Because antibiotic use can interfere with the antibiotic resistance profile of the isolated bacteria pregnant women who were on antibiotic treatment two weeks before their initial visit to the facility and those at 38 weeks or more gestational age were excluded from the study. Other obstetric information was extracted from the antenatal books of pregnant women. Sociodemographic information was obtained using a well-structured questionnaire. All the pregnant women who agreed to written consent were recruited for the study.

### 2.3. Bacteria Culture and Identification

Clean catch mid-stream urine samples were collected from all participants using a wide-mouthed sterile-capped container. The specimen was promptly transported to the microbiology laboratory and cultured within one hour of collection. We used a 0.001 ml calibrated wire loop to inoculate samples onto Cystine Lactose Electrolyte Deficient (CLED) agar, blood agar, and chocolate agar (plates (Oxoid Ltd., Hampshire, United Kingdom) and incubate at 37°C for 24–48 hours. Colony counts yielding bacterial growth of 10^5^/ml of urine were regarded as significant for bacteriuria. Colony characteristics, Gram reaction, and biochemical reactions were used to identify bacterial isolates [29, 30]. For the identification of Gram-negative bacteria, oxidase test, lactose fermentation, and hydrogen sulfur (H_2_S) production in Kligler's iron agar (KIA) test, urease test, citrate test, and indole test were used. Gram positives were also identified using a type of haemolysis, a catalase test, and a coagulase test. Isolated bacteria were later confirmed using API 20E and API 20NE (bioMerieux) for Gram-negative bacteria and API-staph (bioMerieux) and API- Strep (bioMerieux) for *Staphylococcus* and *Streptococcus* species, respectively.

### 2.4. Antimicrobial Susceptibility Test

Antibiotics that are commonly prescribed and are mostly used for empirical treatment were selected for antibiotic susceptibility tests. The test was performed on all isolates according to the Clinical & Laboratory Standards Institute (CLSI) protocol [31]. Bacteria isolates were tested with the following drugs: amoxicillin-clavulanic acid (20 *μ*g), ampicillin (30 *μ*g), chloramphenicol (30 *μ*g) and ceftriaxone (10 *μ*g), cefoxitin (30 *μ*g), ciprofloxacin (5 *μ*g), erythromycin (15 *μ*g), norfloxacin (10 *μ*g), gentamicin (10 *μ*g), nitrofurantoin (50 *μ*g), tetracycline (30 *μ*g), cotrimoxazole (25 *μ*g), and vancomycin (30 *μ*g).

### 2.5. Statistical Analysis

All data were entered into Microsoft office excel 2016 and exported into SPSS version 22 (SPSS Inc., Chicago, IL, USA) for analysis. Data were presented as frequencies and percentages and compared using the chi-square test for categorical values and with the student *t*-test for continuous variables. Univariate and multivariate logistic regression models were performed to determine the association between demographic characteristics and UTIs. A *P* value less than 0.05 was considered statistically significant.

## 3. Results

### 3.1. Participants' Demography and UTI Distribution

Table 1 shows the demographic characteristics and distribution of UTIs among 560 study participants. The overall prevalence of UTI among pregnant women receiving antenatal care was 39.8%, out of which 42/127 (33.1%) were symptomatic and 181/433(41.8%) asymptomatic. Among the 223 pregnant women who had UTIs, 181 (81.2%) had no known history of UTIs. Most of the participants (36.4%) were within 21–25 years of age, had an education to primary school level (46.3%), were unemployed mothers (46.3%), and more than half, 313 (55.9%) had been four to nine times pregnant. More than three-quarters (77.3%) of the participants were asymptomatic for UTI, and 409 (73.0%) had no known history of UTI. Regarding genital hygiene, 129 (23.0%) always wash or wipe their genital area after sex while 308 (54.5%) of the participants do that once in a while. We also observed from the study that a considerable proportion (55.0%) of the participants use public toilet facilities, and the use of paper is the commonest mode of cleaning after defecation. There were statistically significant differences between participants' background characteristics and UTIs except for symptomatic and asymptomatic UTIs (*p*=0.081).

### 3.2. Bacterial Aetiologies Isolated from Urine Culture

**Figure 1:**
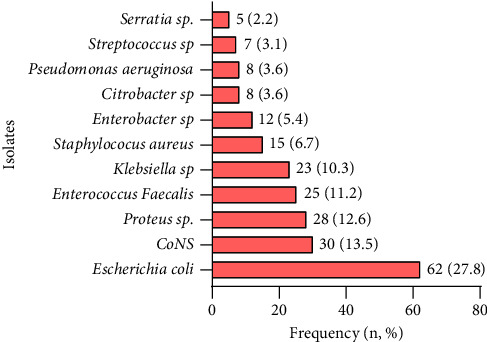
Distribution of bacterial aetiologies isolated from urine culture.

As detailed in Figure 1, the commonest bacteria isolated from the urine cultures were *Escherichia coli* (27.80%) followed by CoNS (13.45%), *Proteus* species (12.6%), *Enterococcus faecalis* (11.2%), *Klebsiella* species (10.3%), *Staphylococcus aureus* (6.7%), *Enterobacter* species (5.4%), *Citrobacter* species (3.6%), *Pseudomonas aeruginosa* (3.6%), *Streptococcus* species (3.1%), and *Serratia* species (2.2%).

Figure 1 shows the frequency distribution of bacterial aetiologies isolated from urine culture among study participants.

### 3.3. Predictors of UTI among Pregnant Women

To identify the predictors of UTI among pregnant women, we performed a multivariate logistic regression analysis of participants' demography with the prevalence of UTI (Table 2). The regression model analysis reveals that pregnant women with gravidity of 4–6, and 6–9 were 2.89 (CI: 1.78–4.67; *p* <  0.0001) and 3.78 (CI: 2.43–5.78; *p*  < 0.0001) times likely to suffer UTI. Also, primiparous women were 1.97 times significantly likely to get a UTI (CI: 1.21–3.65; *p*=0.014). More significantly, those who use public defecation facilities and an individual who practice open defecation were 4.65 (CI: 2.90–9.87: *p* <  0.0001) and 9.45 (CI: 4.89- 17.80; *p* <  0.0001) time more likely to get a UTI. On the other hand, pregnant women within the age range 26–30, 31–35, and ≥36 years recorded a decreasing a significant likelihood of UTI positivity. Participants who frequently [OR = 0.10 (0.06–0.67), *p* < 0.001] practiced genital cleaning after sex were at lower odds of getting a UTI.

### 3.4. Antibiogram Pattern of UTI-Associated Bacterial Isolates

**Figure 2:**
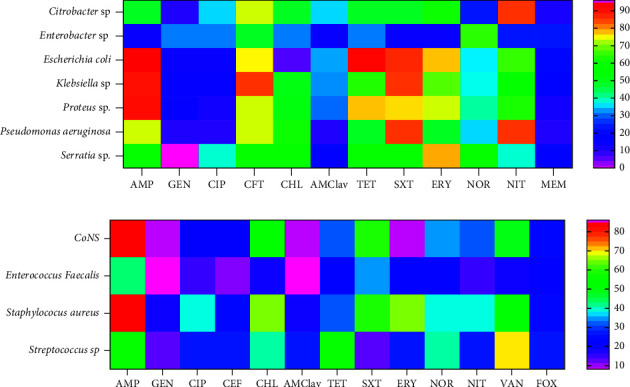
The antibiotic resistance pattern of bacterial isolates among study participants: (a) Antibiotic resistance pattern of Gram-negative isolates; (b) Antibiotic resistance pattern of Gram-positive isolates. AMClav: amoxicillin-clavulanic acid, AMP: ampicillin, SXT: cotrimoxazole, CEF: ceftriaxone, CHL: chloramphenicol, CIP: ciprofloxacin, GEN: gentamicin, ERY: erythromycin, NIT: nitrofurantoin, NOR: norfloxacin, MEM: meropenem and TET: tetracycline.

We investigated the antimicrobial resistance pattern of isolates as shown in Figure 2. Gram-negatives were resistant to ampicillin in 144 (98.6%) of participants, followed by resistance to cotrimoxazole (131, 89.7%), ceftriaxone (123, 84.2%), tetracycline (114, 78.1%), erythromycin (107, 73.3%), nitrofurantoin (98, 67.1%), norfloxacin (61, 41.8%), chloramphenicol (58, 39.7%), and the least to amoxicillin-clavulanic acid (52, 35.6%), respectively. Low resistance was observed for antibiotics such as ciprofloxacin (32, 21.9%), meropenem (29, 19.9%), and gentamicin (29, 19.9%). Gram positives were resistant to ampicillin in 54 (70.1%) of the participants, followed by resistance to erythromycin (45, 58.4%), cotrimoxazole (37, 48.1%), vancomycin (37, 48.1%), chloramphenicol (35, 45.5%), and ceftriaxone (31, 40.3%) in that order. There was low resistance against gentamicin in 9 (11.7%) of the participants, amoxicillin-clavulanic acid in 10 (13.0%), ciprofloxacin in 19 (24.7%), and nitrofurantoin in 22 (28.6%) of the participants, respectively.

We also detected the multidrug resistance pattern of the bacteria isolated. Generally, 206 (92.4%) of the bacteria isolated were resistant to at least one antibiotic, however, 152 (68.2%) of the 223 bacteria isolated were multidrug resistant. Among the bacteria species, *E. coli* 35 (56.5%), *Klebsiella* sp. 18 (78.3%), and *Proteus* sp. 20 (71.4%) dominated the multidrug-resistant (MDR) Gram-negative bacteria, whereas *Staphylococcus aureus* 13 (86.7%) was also found to dominate the multidrug-resistant Gram-positive bacteria (Table 3).

## 4. Discussion

The overall prevalence of UTI among pregnant women in this study was 39.8% which is comparable to the rate of 42.7% reported in a previous study conducted in Ghana [16] and lower than the 56.5% reported in a study conducted in a different setting in southern Ghana [32]. Conversely, the observed UTI prevalence was higher than the earlier reported rate of 17.1% in the Ho Municipality of Ghana [33]. Our present result represents one of the highest UTI prevalences to date compared to previous findings in Africa and Asia. For instance, different studies from different parts of Ethiopia such as Gondar, Addis Ababa, and Dessie have revealed UTI prevalence ranges of 9.5%–15.5% [19, 34, 35]. In addition, lower reported prevalence rates have also been reported in Kenya (15.7%) [36] and Nigeria (15.8% and 25.3%) [37]. Among the other countries are Tanzania (16.8%) [38], Cameroon (23.5%) [39], Iran (13.1%) [37], Nepal (30.5%) [4], and India (20.1%–28.0%) [40, 41]. Nonetheless, the prevalence rate reported in a study conducted in Niger [12] is higher than our result. These findings highlight that UTI prevalence rates vary within a country, across countries and geographical areas, respectively, which can be attributed to factors such as varied personal hygiene practices, attitudes around UTIs, sexual behaviour, limited healthcare infrastructure, and diagnostic tools, as well as risk factors such as age and parity [35, 37, 42–44].

Asymptomatic UTI has been reported to be the commonest form of pregnancy-associated UTI [37]. We observed the same in this study, as the prevalence of UTI among symptomatic pregnant women was 33.0% whereas that of asymptomatic was 41.8%. The observed prevalence of asymptomatic UTI in this study is higher than in earlier studies among pregnant women in the Ghanaian community [24, 32, 45] and in other populations from developing countries like Ethiopia [34, 35, 46, 47], Tanzania [38], and Kenya [36]. These observed differences could be partially explained by the study population and sample sizes, and a lack of infrastructure, and the employment of different approaches for the diagnosis of UTI [34, 35].

Parity and gravidity play a vital role in conferring the infection during pregnancy [5, 44]. In this study, we found that women who have had four or more pregnancies were at increased risk of suffering UTIs. This finding concord with reports from studies conducted in Nigeria [48], Egypt [27], and Iran [49]. Conversely, no association was found between parity and UTI among pregnant women in cross-sectional studies from Sudan [50], Tanzania [23], Ghana [51], and West Ethiopia [52]. Poor hygiene practices during pregnancy have been associated with UTIs among pregnant women [53]. In this study, participants who frequently practice genital cleaning after sex had lower odds of getting a UTI. We also found out that those who use public defecation facilities and individuals who practice open defecation were five and nine times, respectively, more likely to get UTIs. A cross-sectional study by Parasuraman et al. [54] indicated that people who use public toilet facilities are at risk of various bacterial infections. Therefore, the use of the same sanitary facilities by strangers comes with related risks of faecal bacteria transmission [55].

Most of the bacterial isolates from the urine samples of pregnant women in this study were Gram-negative. *E. coli* was the commonest bacteria isolates. A similar trend has been reported in Ghana [13, 16, 24, 32], Nigeria [22], Sudan [50], Libya [56], and Ethiopia [34, 46]. The possible explanation for the predominance of Gram-negative bacteria among isolated UTI aetiological agents may be because they are common members of the vaginal and rectal flora [34, 47, 57]. On the other hand, CoNS dominated the Gram-positive isolates, followed by *Enterococcus faecalis* and *Staphylococcus aureus*. While some studies have detected *CoNS* as the most frequently isolated bacteria [11, 34], others detected *S. aureus* as the most frequently isolated Gram-positive bacteria [22, 23, 34, 58, 59]. *Enterococcus faecalis* has also been reported as the predominant Gram-positive bacterial isolate in other studies [45].

Antibiotic susceptibility test show that most isolates are susceptible to gentamycin. Like other studies, isolates' susceptibility to gentamycin was the highest [6, 11, 19, 20, 34]. High resistance was detected against ampicillin, cotrimoxazole, ceftriaxone, tetracycline, erythromycin, and nitrofurantoin. The irrational use and abuse of broad-spectrum antibiotics due to their affordability and easy access may be the reason for the high resistance to these drugs [13, 58]. The 29 (19.9%) Gram-negative bacteria resistant to meropenem may be a threat to antibiotic options available for the treatment of infections because carbapenems have the highest potency against bacteria. It is for this reason that they are reserved and used for more severe infections or as last-line drugs [60]. Another worrying result is the resistance of some Gram-positive bacteria to vancomycin, a glycopeptide whose emerging resistance is seen among enterococci and staphylococci which has led to restrictive use to only severe infections caused by Gram-positive bacteria for which no other alternative is acceptable [60].

The 68.2% of multidrug resistance detected among the bacteria isolated from the urine of pregnant women was higher than the 57.1% reported in northern Ethiopia [61]. However, this (68.2%) cannot be compared with the reported 77.5% in Uganda [62], 80.4% from Ethiopia by MA Belete [35], 85.5% in Somaliland [46], 96.0% from Kenya [36], and 100% in Nigeria [42].

A major issue worldwide is the rise in antibiotic resistance, especially in developing nations like Ghana. The MDR rates reported in this study and in the aforementioned countries threaten the effective treatment of UTI in pregnant women as there are greater incidences of antibiotic resistance to popular antimicrobial drugs used to treat urinary tract infections in pregnant women [63]. There will be a need for high-quality, expensive antibiotics. Additionally, this may result in a lengthier treatment period and hospitalisation which can take a toll on family or caregivers [64]. Untreated UTIs during pregnancy can lead to obstetric complications as well as poor maternal and neonatal outcomes [65]. Therefore, it is impossible to ignore the teratogenic effect of prolonged antibiotic exposure in pregnant women [66].

Nevertheless, the findings of this study are consistent with reports from numerous studies. This study's cross-sectional design limits its capacity to determine the cause-and-effect link between risk factors and UTI status, which is one of its limitations. Additionally, we did not assess the adverse effect and symptoms of pregnant women with UTIs and other coinfected conditions, underlying the need for further studies to address these limitations.

## 5. Conclusion

The current findings demonstrate that a high prevalence of UTIs exists among pregnant women in the northern part of Ghana. *Escherichia coli* was the most predominant bacteria isolated. UTI in pregnancy was associated with risk factors such as the use of public defecation facilities, open defecation, and a lack of good practice in genital hygiene after sex. The variation in the resistance pattern of isolates to various drugs observed in this study and the consequences of undiagnosed and untreated UTIs underpin the need to perform urine culture and antibiotic susceptibility before treatment.

## Figures and Tables

**Table 1:** Background characteristics and distribution of UTI among study participants.

Variables	Numbers tested for UTI							
	Total *N* = 560		Positive (*n* = 223)		Negative (*n* = 337)		*X* ^2^, df	*P*-value
	*n*	(%)	*n*	(%)	*n*	(%)		
*Age groups (years)*							40.5, 4	<0.0001
<20	104	(18.6)	48	(46.2)	56	(53.8)		
21–25	204	(36.4)	98	(48.0)	106	(52.0)		
26–30	128	(22.9)	58	(45.3)	70	(54.7)		
31–35	82	(14.6)	14	(17.1)	68	(82.9)		
>36	42	(7.5)	5	(11.9)	37	(88.1)		

*Education*							73.2, 4	<0.0001
None	122	(21.8)	87	(71.3)	35	(28.7)		
Primary	259	(46.3)	72	(27.8)	187	(72.2)		
JHS	126	(22.5)	52	(41.3)	74	(58.7)		
SHS	51	(9.1)	12	(23.5)	39	(76.5)		
Tertiary	2	(0.4)	0	(0.0)	2	(100.0)		

*Occupation*							17.5, 4	0.0016
None	259	(46.3)	107.0	(41.3)	152	(58.7)		
Trading	102	(18.2)	38.0	(37.3)	64	(62.7)		
Apprenticeship	62	(11.1)	18.0	(29.0)	44	(71.0)		
Farming	124	(22.1)	68.0	(54.8)	56	(45.2)		
Formal	13	(2.3)	2.0	(15.4)	11	(84.6)		

*Gravidity*							32.8, 3	<0.0001
1–3	151	(27.0)	38	(25.2)	113	(74.8)		
4–6	185	(33.0)	89	(48.1)	96	(51.9)		
6–9	128	(22.9)	68	(53.1)	60	(46.9)		
>9	96	(17.1)	28	(29.2)	68	(70.8)		

*Parity*							11.7, 2	0.0029
Nullipara	128	(22.9)	46	(35.9)	82	(64.1)		
Primipara	154	(27.5)	79	(51.3)	75	(48.7)		
Multipara	278	(49.6)	98	(35.3)	180	(64.7)		

*Trimester*							21.4, 2	<0.0001
First	185	(33.0)	97	(52.4)	88	(47.6)		
Second	255	(45.5)	78	(30.6)	177	(69.4)		
Third	120	(21.4)	48	(40.0)	72	(60.0)		

*UTI*								0.0806
Symptomatic	127	(22.7)	42	(33.1)	85	(66.9)		
Asymptomatic	433	(77.3)	181	(41.8)	252	(58.2)		

*History of UTI*								0.0004
Yes	151	(27.0)	42	(27.8)	109	(72.2)		
No	409	(73.0)	181	(44.3)	228	(55.7)		

*Genital hygiene*							123.5, 2	<0.0001
No	126	(22.5)	97	(77.0)	29	(23.0)		
Once a while	305	(54.5)	114	(37.4)	191	(62.6)		
Always	129	(23.0)	12	(9.3)	117	(90.7)		

*Defecation facility*							51.8, 2	<0.0001
Private	113	(20.2)	14	(12.4)	99	(87.6)		
Public	308	(55.0)	131	(42.5)	177	(57.5)		
Open	139	(24.8)	78	(56.1)	61	(43.9)		

*Mode of cleaning after defecation*							83.7, 3	<0.0001
Water from Buta	155	(27.7)	108	(69.7)	47	(30.3)		
Tissue paper	112	(20.0)	31	(27.7)	81	(72.3)		
Paper	235	(42.0)	63	(26.8)	172	(73.2)		
Others	58	(10.4)	31	(53.4)	27	(46.6)		

UTI, urinary tract infection; *X*^2^, Chi-square value; df, degree of freedom; *p* < 0.001, statistically significant.

**Table 2:** Logistic regression analysis of demographic characteristics with the prevalence of UTI.

Variables	cOR	95% CI	*P* value	aOR	95% CI	*P* value
*Age groups (years)*						
<20	1			1		
21–25	1.08	0.68–1.73	0.809	1.28	0.67–1.76	0.814
26–30	0.97	0.57–1.64	0.999	0.90	0.61–1.72	0.878
31–35	0.24	0.12–0.48	<0.0001	0.19	0.14–0.54	<0.0001
>36	0.16	0.06–0.41	<0.0001	0.17	0.07–0.46	<0.0001

*Education*						
None	1			1		
Primary	0.15	0.09–0.26	<0.0001	0.13	0.08–0.32	<0.0001
JHS	0.28	0.17–0.48	<0.0001	0.32	0.20–0.52	<0.0001
SHS	0.12	0.06–0.26	<0.0001	0.17	0.10–0.34	<0.0001

*Occupation*						
None	1			1		
Trading	0.84	0.52–1.36	0.551	0.98	0.54–1.75	0.516
Apprenticeship	0.58	0.32–1.07	0.083	0.64	0.34–1.45	0.090
Farming	1.73	1.12–2.64	0.016	1.8	1.21–2.75	0.010
Formal	0.26	0.06–1.09	0.082	0.28	0.08–1.16	0.080

*Gravidity*						
1–3	1			1		
4–6	2.76	1.73–4.44	<0.0001	2.89	1.78–4.67	<0.0001
6–9	3.37	2.01–5.66	<0.0001	3.78	2.43–5.78	<0.0001
>9	1.22	0.69–2.13	<0.0001	1.36	0.82–2.54	<0.0001

*Parity*						
Nullipara	1			1		
Primipara	1.88	1.17–3.07	0.012	1.97	1.21–3.65	0.014
Multipara	0.97	0.63–1.50	0.911	0.98	0.78–1.89	0.914

*Trimester*						
First	1			1		
Second	0.40	0.27–0.59	<0.0001	0.41	0.26–0.87	<0.0001
Third	0.60	0.38–0.97	0.035	0.63	0.37–0.94	0.036

*UTI*						
Symptomatic	1			1		
Asymptomatic	1.45	0.96–2.23	0.081	1.54	0.97–2.65	0.087

*History of UTI*						
Yes	1			1		
No	2.06	1.37–3.11	0.0004	2.54	1.65–3.15	0.0006

*Genital hygiene (wash/wipe after sex)*						
No	1			1		
Once a while	0.18	0.11–0.28	<0.0001	0.20	0.15–0.20	<0.0001
Always	0.03	0.02–0.06	<0.0001	0.10	0.06–0.67	<0.0001

*Defecation facility*						
Private	1			1		
Public	5.23	2.86–9.50	<0.0001	4.65	2.90–9.87	<0.0001
Open	9.04	4.76–17.10	<0.0001	9.45	4.89–17.34	<0.0001

*Mode of cleaning after def.*						
Water from Buta	1			1		
Tissue paper	0.16	0.10–0.29	<0.0001	0.20	0.14–0.32	<0.0001
Paper	0.15	0.10–0.25	<0.0001	0.45	0.04–0.89	<0.0001
Others	0.5	0.27–0.92	0.035	0.64	0.20–0.98	0.067

aOR = adjusted odds ratio; cOR = crude odds ratio; CI, confidence interval; UTI, urinary tract infections; *p* < 0.001, statistically significant.

**Table 3:** Multidrug resistance profile of isolated bacteria.

Bacteria	*N*	*R*0		*R*1		*R*2		*R*3		*R*4		*R*5		*R*6		*R*7		MDR	
		*n*	(%)	*n*	(%)	*n*	(%)	*n*	(%)	*n*	(%)	*n*	(%)	*n*	(%)	*n*	(%)	*n*	(%)
CoNS	30	4	(13.3)	4	(13.3)	2	(6.7)	5	(16.7)	8	(26.7)	5	(16.7)	2	(6.7)	0	(0.0)	20	(66.7)
*Enterococcus Faecalis*	25	2	(8.0)	0	(0.0)	0	(0.0)	3	(12.0)	4	(16.0)	4	(16.0)	2	(8.0)	1	(4.0)	14	(56.0)
*Staphylococcus aureus*	15	0	(0.0)	0	(0.0)	2	(13.3)	5	(33.3)	3	(20.0)	3	(20.0)	2	(13.3)	0	(0.0)	13	(86.7)
*Streptococcus* sp.	7	0	(0.0)	0	(0.0)	0	(0.0)	2	(28.6)	1	(14.3)	3	(42.9)	0	(0.0)	0	(0.0)	6	(85.7)
*Citrobacter* sp.	8	1	(12.5)	0	(0.0)	0	(0.0)	2	(25.0)	3	(37.5)	2	(25.0)	0	(0.0)	0	(0.0)	7	(87.5)
*Enterobacter* sp.	12	0	(0.0)	0	(0.0)	2	(16.7)	1	(8.3)	2	(16.7)	6	(50.0)	1	(8.3)	0	(0.0)	10	(83.3)
*Escherichia* coli	62	5	(8.1)	12	(19.4)	10	(16.1)	6	(9.7)	8	(12.9)	13	(21.0)	5	(8.1)	3	(4.8)	35	(56.5)
*Klebsiella* sp.	23	0	(0.0)	2	(8.7)	3	(13.0)	5	(21.7)	6	(26.1)	7	(30.4)	0	(0.0)	0	(0.0)	18	(78.3)
*Proteus* sp.	28	2	(7.1)	4	(14.3)	2	(7.1)	5	(17.9)	7	(25.0)	5	(17.9)	2	(7.1)	1	(3.6)	20	(71.4)
*P. aeruginosa*	8	2	(25.0)	0	(0.0)	0	(0.0)	4	(50.0)	2	(25.0)	1	(12.5)	0	(0.0)	0	(0.0)	7	(87.5)
*Serratia* sp.	5	1	(20.0)	0	(0.0)	2	(40.0)	2	(40.0)	0	(0.0)	0	(0.0)	0	(0.0)	0	(0.0)	2	(40.0)
	223	17	(7.6)	23	(10.3)	25	(11.2)	43	(19.3)	48	(21.5)	54	(24.2)	20	(9.0)	12	(5.4)	152	(68.2)

*Note. R*0, no antibiotic resistance; *R*1, resistance to one; *R*2, resistance to two; *R*3, resistance to three; *R*4, resistance to four; *R*5, resistance to five; *R*6, resistance to six; *R*7, resistance to seven; MDR, multidrug-resistant.

## Data Availability

The data will be made available upon reasonable request through the corresponding author.
